# Physiological concentration of protocatechuic acid directly protects vascular endothelial function against inflammation in diabetes through Akt/eNOS pathway

**DOI:** 10.3389/fnut.2023.1060226

**Published:** 2023-03-21

**Authors:** Chui Yiu Bamboo Chook, Yiu Ming Cheung, Ka Ying Ma, Fung Ping Leung, Hanyue Zhu, Qingshan Jason Niu, Wing Tak Wong, Zhen-Yu Chen

**Affiliations:** ^1^School of Life Sciences, The Chinese University of Hong Kong, Hong Kong, Hong Kong SAR, China; ^2^School of Food Science and Engineering, Foshan University, Foshan, Guangdong, China; ^3^Institute for Advanced Study, Shenzhen University, Shenzhen, Guangdong, China

**Keywords:** protocatechuic acid, diabetes mellitus, endothelial function, anti-inflammatory, vascular endothelial cell

## Abstract

**Background:**

Cardiovascular diseases (CVDs) have been the major cause of mortality in type 2 diabetes. However, new approaches are still warranted since current diabetic medications, which focus mainly on glycemic control, do not effectively lower cardiovascular mortality rate in diabetic patients. Protocatechuic acid (PCA) is a phenolic acid widely distributed in garlic, onion, cauliflower and other plant-based foods. Given the anti-oxidative effects of PCA *in vitro*, we hypothesized that PCA would also have direct beneficial effects on endothelial function in addition to the systemic effects on vascular health demonstrated by previous studies.

**Methods and results:**

Since IL-1β is the major pathological contributor to endothelial dysfunction in diabetes, the anti-inflammatory effects of PCA specific on endothelial cells were further verified by the use of IL-1β-induced inflammation model. Direct incubation of *db/db* mouse aortas with physiological concentration of PCA significantly ameliorated endothelium-dependent relaxation impairment, as well as reactive oxygen species overproduction mediated by diabetes. In addition to the well-studied anti-oxidative activity, PCA demonstrated strong anti-inflammatory effects by suppressing the pro-inflammatory cytokines MCP1, VCAM1 and ICAM1, as well as increasing the phosphorylation of eNOS and Akt in the inflammatory endothelial cell model induced by the key player in diabetic endothelial dysfunction IL-1β. Upon blocking of Akt phosphorylation, p-eNOS/eNOS remained low and the inhibition of pro-inflammatory cytokines by PCA ceased.

**Conclusion:**

PCA exerts protection on vascular endothelial function against inflammation through Akt/eNOS pathway, suggesting daily acquisition of PCA may be encouraged for diabetic patients.

## 1. Introduction

Protocatechuic acid (PCA) is a phenolic compound commonly found in a wide range of spices, including garlic, onion, star anise, olive and chicory; vegetables, such as cauliflower and bitter melon; and as a primary metabolite of anthocyanins in human intestines (1, 2). Increasing attention has recently been drawn toward the health-promoting effects of PCA due to its anti-oxidative, anti-inflammatory, anti-ageing and anti-carcinogenic properties against a wide range of diseases, including neurodegenerative disease, cancers, diabetes and cardiovascular diseases (CVDs) (3).

A study has reported that 6-week oral administration of PCA in streptozotocin-induced diabetic rats lead to restoration in fasting blood glucose, blood insulin, blood pressure and plasma antioxidant enzyme levels (4). In particular, the mean arterial pressure in the diabetic rats in response to the vasodilator acetylcholine (ACh) was rescued, providing direct evidence on the cardiovascular benefits of PCA (4). Another research showed that oral intake of PCA for 12 weeks improved vasodilation induced by insulin and insulin-like growth factor-1 (IGF-1) in aged spontaneously hypertensive rats (5). The anti-oxidative effects of PCA on palmitic acid (PA)-treated vascular endothelial cells had also been reported (6, 7), reinforcing the vasoprotective activity of PCA.

Endothelial cells are the innermost single layer of cells in blood vessels (8). They are responsible for the maintenance of vascular homeostasis, including regulation of smooth-muscle tone, oxidative stress and inflammation by secreting various factors such as prostacyclin (PGI_2_), endothelin-1 and nitric oxide (NO). NO is involved in not only the relaxation of smooth muscle or vasodilation, but also the modulation of oxidative stress and inflammation in vasculature (8). The reduction in NO bioavailability resulting in the deprivation of regulatory mechanisms in endothelial cells is a key characteristic of endothelial dysfunction, which can further lead to atherosclerosis and other CVDs (9). Nonetheless, the effects of PCA, if any, on these factors remain largely unclear.

Diabetic patients are particularly susceptible to endothelial dysfunction due to the mixed effects of hyperglycemia, oxidative stress, as well as the chronic inflammatory condition built by the vicious cycle of pro-inflammatory cytokine signaling (10). This complex pathology of diabetic endothelial dysfunction not only contributes to the high mortality rate due to CVDs in diabetic patients, but also the inability for classic glycemic control medications to effectively reduce cardiovascular events (11). While previous studies have suggested that oral PCA administration can protect vascular health against oxidative damage by upregulating blood anti-oxidative enzyme levels, there is a lack of evidence to assess if PCA has a direct beneficial effect on endothelial function in diabetes. Moreover, it is also unknown whether PCA exerts protection against vascular inflammation, which is the major pathological mechanism in diabetic vascular diseases. Therefore, the present study aims to see if direct incubation of PCA could protect vascular function against inflammation in diabetes.

## 2. Materials and methods

### 2.1. Experimental animals

All animals were supplied by the Laboratory Animal Service Center (LASEC), the Chinese University of Hong Kong (CUHK) with the approval by the Animal Experimentation Ethics Committee (AEEC), CUHK. Animals used in the present study included male and female type 2 diabetic *db/db* mice lacking functional leptin receptors from C57BL/KSJ background (12), the counterpart heterozygote *db/m^+^* mice and C57BL/6J mice. The mice were housed in a temperature-controlled holding room (22–24°C) with a 12-h light/dark cycle. Standard chow diet and water were provided *ad libitum*.

### 2.2. Aortic ring preparation for functional studies

After the mice were euthanized, their thoracic aortas were rapidly excised and immersed in ice-cold phosphate-buffered saline (PBS; cat# 10010023). The surrounding connective tissues and perivascular adipose tissues were removed. The aortas were then cut into 2-mm-long segments with blood clots inside the artery eliminated. The aorta segments were incubated in Dulbecco’s Modified Eagle’s Medium (DMEM; Gibco, MD, USA; cat# 11885084) with 1 g/L glucose, 10% fetal bovine serum (FBS, Gibco; cat# 10270106), 100 IU/mL penicillin and 100 μg/mL streptomycin (Penicillin-Streptomycin, Gibco; cat# 15140122), with or without treatments of IL-1β and protocatechuic acid (10 nM and 100 nM) overnight in a CO_2_ incubator at 37°C overnight.

### 2.3. Vasorelaxation activity by wire myograph

Vasorelaxation activity was measured by changes in isometric tension of the aortic rings which were recorded by the Multi Myograph System (Danish Myo Technology, Aarhus, Denmark) as previously mentioned (13). Each aortic ring was mounted to one chamber filled with 37°C Krebs solution on the Multi Myograph System using two wires and stretched to an optimal baseline tension of 3 mN (monitored in real-time). The aortic rings were allowed to equilibrate in the chamber for 60 mins before 60 mmol/L KCl-containing Krebs solution was added to induce contraction of the aortic rings, which were then rinsed by Krebs solution to restore the baseline tension. Following the contraction induced by 3 μM phenylephrine (Phe), endothelium-dependent relaxation (EDR) was induced along the cumulative addition of acetylcholine (ACh) (3 × 10^–9^ to 10^–5^ M) (14). The aortas were then incubated with L-N*^G^*-nitro-L-arginine methyl ester (L-NAME) for 15 min, which inhibits NO synthase, to examine the endothelium-independent relaxation in a similar manner as EDR with cumulative addition of sodium nitroprusside (SNP) (10^–9^ to 10^–5^ M) instead.

### 2.4. Endothelial cell culture

Mouse brain microvascular endothelial cells (mBMECs) (Angio-Proteomie, Boston, MA, USA) were cultured in DMEM (Gibco) with 4.5 g/L glucose, 10% fetal bovine serum (FBS Gibco) and 100 IU/mL penicillin and 100 μg/mL streptomycin (Penicillin-Streptomycin, Gibco), with or without IL-1β (0.5 ng/mL) treatment, PCA (1–100 nM) and Akt inhibitor IV (1 μM) overnight at 37°C in a CO_2_ incubator.

### 2.5. Detection of intracellular reactive oxygen species (ROS) by dihydroethidium fluorescence

The dissected aortas were put into optimal cutting temperature compound (O.C.T.) (Tissue-Tek^®^ O.C.T.™ Compound, Sakura Finetek Europe B.V., Alphen aan den Rijn, Netherland; cat# 4583), which were then snap frozen in liquid nitrogen for embedding. The embedded aortas were cut into slices of 5 μm thick for staining with dihydroethidium (DHE) (Invitrogen, Waltham, MA, USA; cat# D23107) solution, which is blue in the cytosol and turns red when oxidized and intercalating with the nucleus, according to the official protocol provided by the kit (15). Following the staining with 5 μM DHE solution for 30 min with subsequent washing, the emission of red light was detected by confocal microscope (TCS SP8 MP, Leica, Wetzlar, Germany). The total DHE-emitted light intensity (605 nm) was normalized by the autofluorescence area (488 nm) from each aortic ring.

### 2.6. Quantitative polymerase chain reaction (qPCR)

RNA was extracted from homogenized mBMEC samples using RNAiso Plus (TaKaRa, Kyoto, Japan; cat# 9109). RNA was reverse transcribed into cDNA with the use of PrimeScript™ RT Master Mix (TaKaRa; cat# RR036B). Quantitative PCR (qPCR) experiments were performed on the CFX96 Touch™ Real-time PCR Detection System (Bio-Rad, Hercules, CA, USA) with TB Green^®^ Premix Ex Taq™ (Tli RNase H Plus) (TaKaRa; cat# RR420A). Relative expression levels of mRNAs were calculated in relative to 36B4 as the housekeeping gene using the 2^(-Delta Delta C(T)) Method. Primers used for RT-PCR analysis are included in the Supplementary Data.

### 2.7. Western blotting

Protein samples collected from mBMECs homogenates were electrophoresed through a 10% SDS-polyacrylamide gel and transferred onto an Immun-Blot^®^ PVDF membrane (Bio-Rad; cat# 1620177). Non-specific binding was blocked by 3% BSA in 0.1% Tween-20 TBS for 1 hour. The blots were then incubated overnight at 4°C with primary antibodies: anti-phospho-eNOS Ser1177 (1:1,000, Cell Signaling Technology, CST, Danvers, MA, USA; cat# 9570S), anti-eNOS (1:1,000, CST; cat# 32027S), anti-phospho-Akt Ser473 (1:1,000, CST; cat# 9271S), anti-Akt (1:1,000, CST; cat# 9272S), MCP1 (1:1,000, CST; cat# 2027), GAPDH (1:5000, CST; cat# M00227). The blots were eventually incubated with goat anti-rabbit IgG (H + L) horseradish peroxidase conjugate (1:10,000, Invitrogen; cat# G21234) prior to chemiluminescence detection by the ChemiDoc MP Imaging System (Bio-Rad) with SuperSignal™ West Pico PLUS Chemiluminescent Substrate (Thermo Fisher Scientific).

### 2.8. Drugs and solutions

Protocatechuic acid (PCA) was purchased from Sigma-Aldrich Chemical (St Louis, MO, USA; cat# 37580-100g-F) and dissolved in PBS. IL-1β was purchased from PeproTech (Rocky Hill, NJ, USA; cat# 211-11B/10UG). Akt inhibitor IV was purchased from MilliporeSigma (Billerica, MA, USA; cat# 124011-1MG). Acetylcholine (ACh; cat# A6625-25G), N^G^-nitro-L-arginine methyl ester (L-NAME; cat# N5501-5G), phenylephrine (Phe; cat# P6126-10G) and sodium nitroprusside (SNP; cat# 71778-25G) were purchased from Sigma-Aldrich Chemical and dissolved in double-distilled water. Krebs-Henseleit solution is composed of (mM): 119 NaCl, 4.7 KCl, 2.5 CaCl_2_, 1 MgCl_2_, 25 NaHCO_3_, 1.2 KH_2_PO_4_, and 11 D-glucose.

### 2.9. Statistical analysis

Results were expressed as means ± SEM for each group. Statistical significance was determined by two-tailed Student’s *t*-test, one-way or two-way ANOVA when appropriate using GraphPad Prism software (Version 8.0, San Diego, CA, USA). A Bonferroni correction was performed for multiple comparisons. *P* < 0.05 was regarded as statistically significant.

## 3. Results

### 3.1. Direct incubation with protocatechuic acid (PCA) improved the impaired endothelium-dependent relaxation (EDR) in both male and female *db/db* mouse aortas with ROS suppression

**FIGURE 1:**
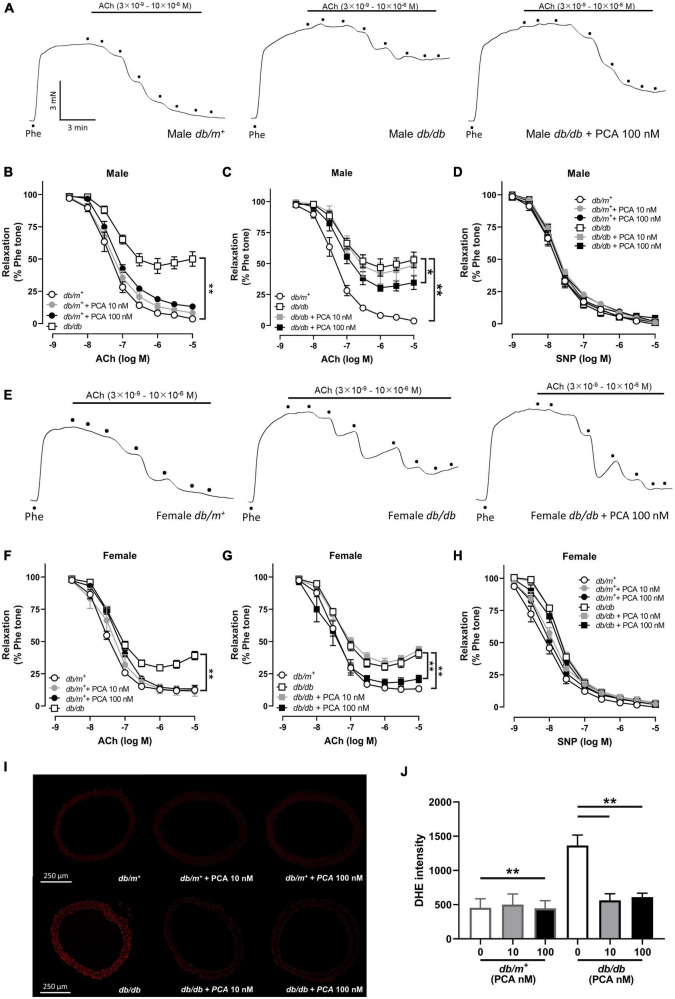
Direct PCA incubation improved the endothelium-dependent relaxation (EDR) impairment in both male and female db/db mouse aortas with ROS suppression. Representative tracings **(A)** with summarized data **(B,C)** of EDR and endothelium-independent relaxation **(D)** measured by wire myograph in aortas from male *db/m^+^* and *db/db* mice incubated with and without PCA (10 – 100 nM). Representative tracings **(E)** with summarized data **(F,G)** of EDR and endothelium-independent relaxation **(H)** measured by wire myograph in aortas from female *db/m^+^* and *db/db* mice incubated with and without PCA (10 – 100 nM). Representative confocal images **(I)** and summarized data **(J)** of DHE stain intensity in aortic rings from *db/m^+^* and *db/db* mice with and without PCA incubation (10–100 nM). Data are presented in means ± SEM; *n* = 5; *p < 0.05, **p < 0.01. PCA, protocatechuic acid; Phe, phenylephrine; ACh, acetylcholine; SNP, sodium nitroprusside; DHE, dihydroethidium.

First, we aimed to determine if PCA could reverse the endothelial dysfunction in diabetic mouse aortas. In consistence to the previous studies, aortas from *db/db* mice showed a stronger contraction, but not significantly, than *db/m^+^* mice, while both 10-nM and 100-nM PCA treatment did not affect the contraction of aortas (Figures 1A, E and Supplementary Figure 1) (16). In both males (Figures 1A–D) and females (Figures 1E–H), endothelium-dependent relaxation (EDR) in *db/db* mouse aortas was reduced compared to *db/m^+^*. While the aortas from *db/m^+^* were not affected (Figures 1B, F), co-incubation with 10 nM and 100 nM of PCA dose-dependently reversed the impaired EDR in both male and female *db/db* mouse aortas (Figures 1C, G). Upon the blockage of nitric oxide (NO) synthase by L-NAME, the endothelium-independent relaxation induced by SNP showed no statistical difference among all groups (Figures 1D, H). Moreover, diabetic *db/db* mouse aortas showed a heightened production of reactive oxygen species (ROS) as compared with *db/m^+^*, which was reduced by co-incubation with 10 nM and 100 nM PCA (Figures 1I, J).

### 3.2. PCA ameliorated the IL-1β-induced EDR impairment and ROS overproduction in C57BL/6J mouse aortas

**FIGURE 2:**
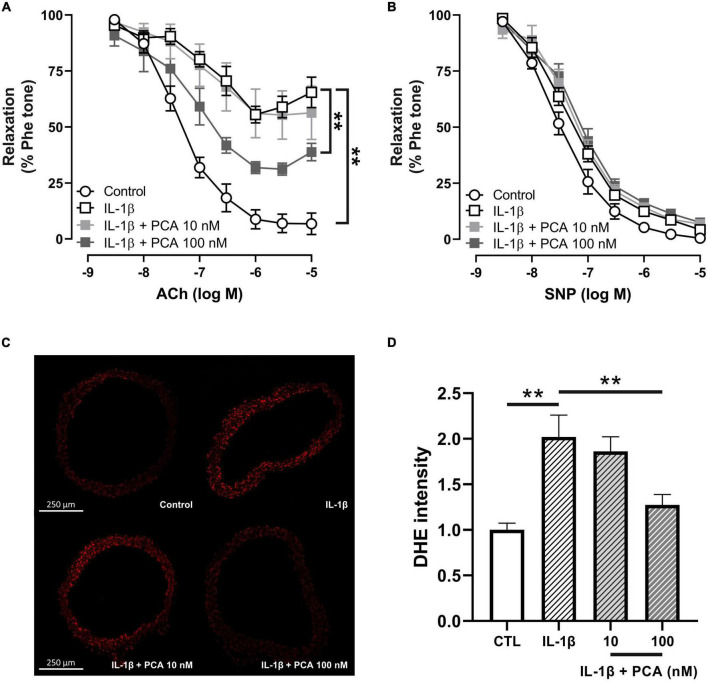
PCA alleviated the impaired EDR and ROS overproduction in C57BL/6J mouse aortas treated with IL-1β. Summarized data **(A)** of EDR and endothelium-independent relaxation **(B)** measured by wire myograph in IL-1β-treated aortas from male C57BL/6J mice incubated with and without PCA (10 – 100 nM); n = 5. Representative confocal images **(C)** and summarized data **(D)** of DHE stain intensity in aortic rings from C57BL/6J mice with and without PCA incubation (10- 100 nM); *n* = 6-7. Data are presented in means ± SEM; **p < 0.01. PCA, protocatechuic acid; Phe, phenylephrine; ACh, acetylcholine; SNP, sodium nitroprusside; DHE, dihydroethidium.

Upregulated level of interleukin 1 beta (IL-1β), which leads to excessive oxidative stress and pro-inflammatory cytokine production, is one of the key players in the pathology of diabetic endothelial dysfunction (17–19). Consistent with previous studies (19), IL-1β induced endothelial dysfunction in non-pathological aortas from C57BL/6 mice (Figure 2A). PCA in 100 nM, however, reversed the dysfunctional EDR (Figure 2A), without affecting the endothelium-independent relaxation (Figure 2B). Furthermore, elevated ROS levels stimulated by IL-1β in C57BL/6 mouse aortas were also decreased by PCA in a dose-dependent manner (Figures 2C, D).

### 3.3. PCA reversed the exaggerated ROS and pro-inflammatory cytokine expression levels stimulated by IL-1β in endothelial cells

**FIGURE 3:**
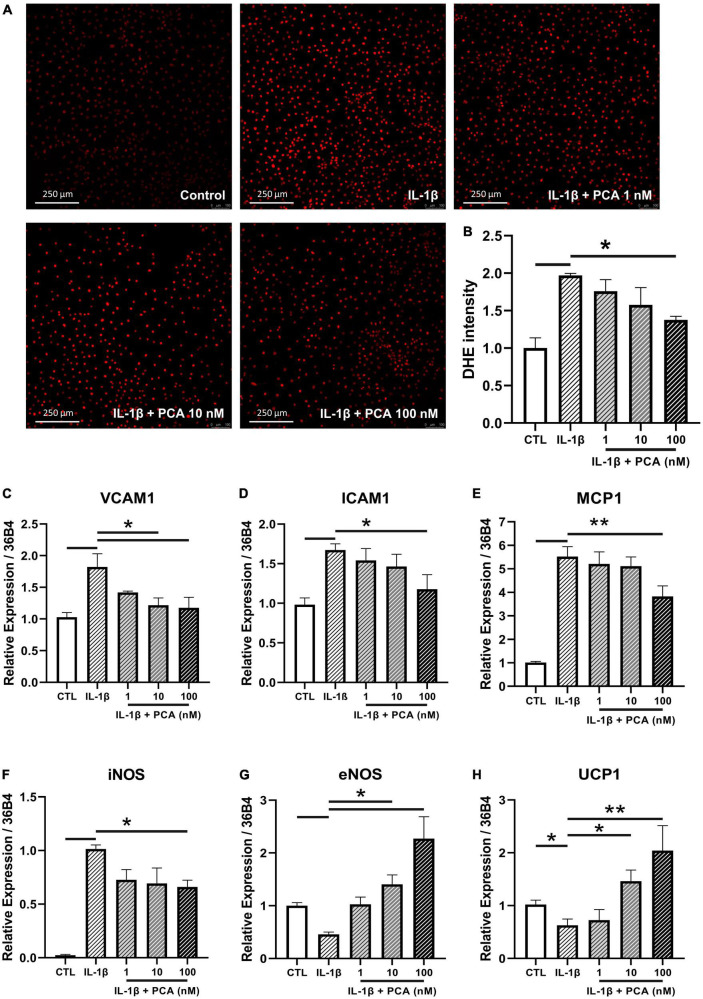
PCA decreased the IL-1β-stimulated ROS and pro-inflammatory cytokine levels in endothelial cells. Representative confocal images **(A)** and summarized data **(B)** of DHE stain intensity in mBMECs with and without PCA incubation (1-100 nM); *n* = 3. Summarized quantification of mRNA levels of VCAM1 **(C)**, ICAM1 **(D)**, MCP1 **(E)**, iNOS **(F)**, eNOS **(G)** and UCP1 **(H)** relative to 36B4 in mBMECs with and without IL-1β (0.5 ng/mL) and PCA (1–100 nM) incubation; *n* = 6–7. Data are presented in means ± SEM; *p < 0.05, **p < 0.01. PCA, protocatechuic acid.

Due to the fact that aorta is composed of different cell types, endothelial cell culture was used to examine the cell-specific effects of PCA. In agreement with the previous measurements in aortic rings, the ROS production triggered by IL-1β in endothelial cells was abolished by PCA treatment dose-dependently, with statistical significance at 100 nM (Figures 3A, B). The mRNA expression levels of pro-inflammatory cytokines, vascular cell adhesion molecule 1 (VCAM1), intercellular adhesion molecule 1 (ICAM1) and monocyte chemoattractant protein-1 (MCP1), as well as the oxidative inducible nitric oxide synthase (iNOS) amplified by IL-1β were abated by PCA in a dose-dependent manner with a peak effect at 100 nM (Figures 3C–F). On the other hand, the vasoprotective endothelial NO synthase (eNOS) and UCP1 were restored by 100 nM PCA (Figures 3G, H). Since GAPDH is involved in the process of glucose metabolism, it has been proposed that the gene expression level of GAPDH would be fluctuated between diabetic and non-diabetic samples (20, 21). Therefore, 36B4, was used instead to normalize the qPCR results (22). At the same time, however, the qPCR data were also validated by GAPDH normalization which resulted in consistent results.

### 3.4. PCA suppressed MCP1 protein expression while restoring eNOS and Akt phosphorylation in IL-1β-treated endothelial cells

**FIGURE 4:**
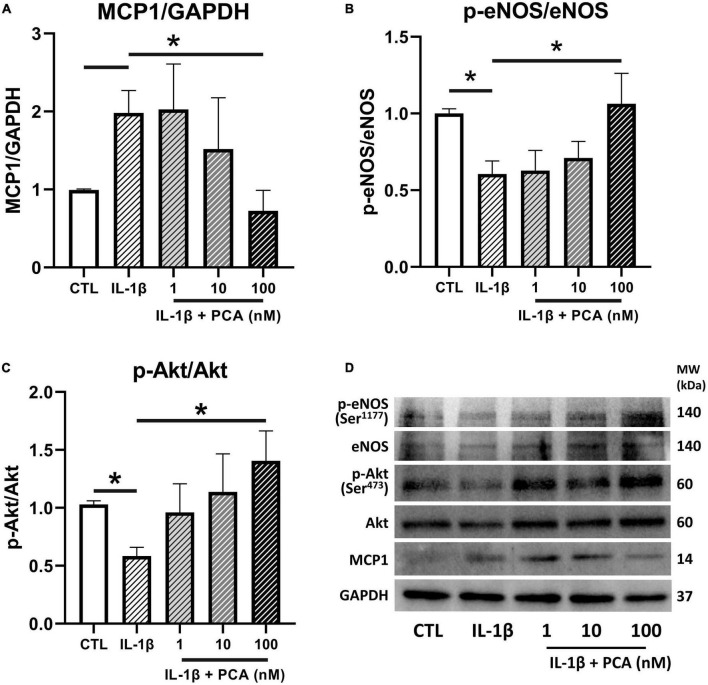
PCA inhibited MCP1 but promoted p-eNOS/eNOS and p-Akt/Akt protein expressions in IL-1β-treated endothelial cells. Summarized protein quantification of **(A)** MCP1, **(B)** p-eNOS/eNOS and **(C)** p-Akt/Akt relative to GAPDH with and without IL-1β (0.5 ng/mL) and PCA (1-100 nM) incubation with representative immunoblots **(D)**; *n* = 6-7. P-eNOS, eNOS, 140 kDa; p-Akt, Akt, 60 kDa; MCP1, 25 kDa; GAPDH, 37 kDa. Data are presented in means ± SEM; *p < 0.05. PCA, protocatechuic acid.

The effects of PCA on the expressions of the pro-inflammatory chemokine MCP1 and the vital endothelial regulator eNOS were further confirmed in protein levels by western blotting. It was consistently found that the MCP1 expressions heightened by IL-1β was downregulated (Figures 4A, D) whereas the inhibited phosphorylation of eNOS was reversed by increasing doses of PCA, resulting in a significant change at 100 nM (Figures 4B, D). In addition, the phosphorylation of the signaling cytokine Akt (Ser^473^), which is involved in the activation of eNOS, was also suppressed by IL-1β but promoted by PCA in a dose-dependent manner (Figures 4C, D). Although p-Akt (Thr^308^) is also involved in eNOS phosphorylation, PCA did not have observable effect on its relative gene expression (Supplementary Figure 2).

### 3.5. Inhibition of akt phosphorylation hindered the anti-inflammatory effects of PCA on endothelial cells

**FIGURE 5:**
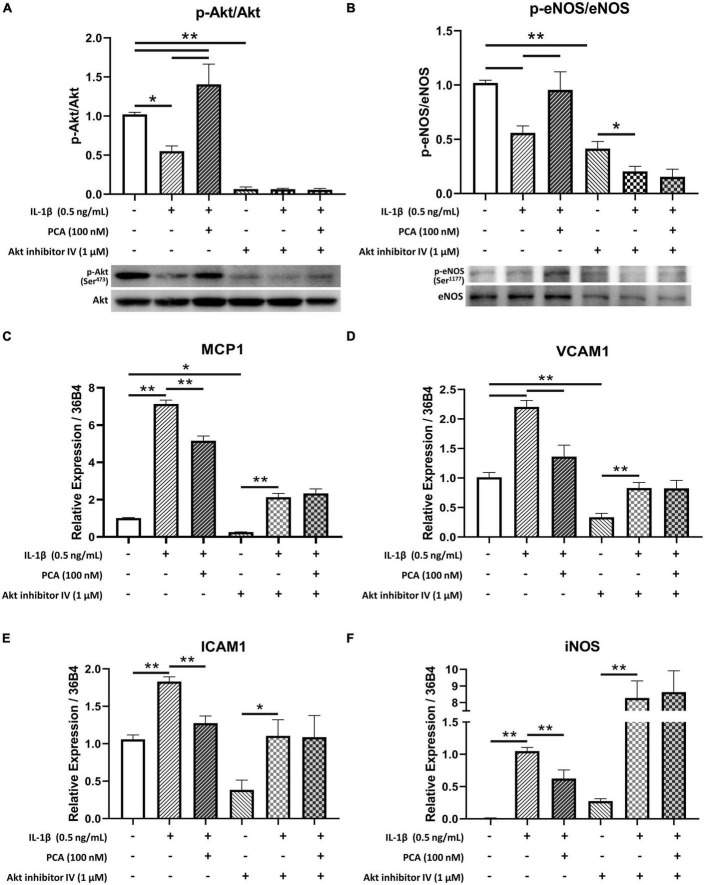
Akt inhibitor IV hindered the restoration of p-eNOS/eNOS and suppression of pro-inflammatory cytokine expressions by PCA in IL-1β-treated endothelial cells. Summarized protein quantification of **(A)** p-Akt/Akt and **(B)** p-eNOS/eNOS relative to GAPDH with and without IL-1β (0.5 ng/mL), PCA (100 nM) and Akt inhibitor IV (1 μM) incubation with representative immunoblots **(D)**; *n* = 5. Summarized quantification of mRNA levels of MCP1 **(C)**, VCAM1 **(D)**, ICAM1 **(E)**, iNOS **(F)** relative to 36B4 in mBMECs with and without IL-1β (0.5 ng/mL), PCA (100 nM) and Akt inhibitor IV (1 μM) incubation; n = 5 – 6. P-eNOS, eNOS, 140 kDa; p-Akt, Akt, 60 kDa; GAPDH, 37 kDa. Data are presented in means ± SEM; *p < 0.05, **p < 0.01. PCA, protocatechuic acid.

Akt inhibitor IV, which inhibits the phosphorylation of Akt (23) (Figure 5A), was used to examine the role of Akt in the effects of PCA on endothelial cells. In the presence of Akt inhibitor, 100 nM PCA failed to rescue the p-eNOS/eNOS ratio in endothelial cells (Figure 5B). The restoration of pro-inflammatory cytokines MCP1, VCAM1 and ICAM1 and oxidative iNOS expression levels was also abolished when Akt phosphorylation was inhibited (Figures 5C–F).

## 4. Discussion

As a minor part of human diet, PCA is either obtained from plant-based foods or produced as a gut microbial metabolite from pigment anthocyanins of color fruits and vegetables (24). PCA has been overlooked in the past due to its relatively low concentration in foods (25). However, PCA has gained more attention after it was found to be the major metabolite of cyanidin-glucosides (CyG) anthocyanins (1). Quantitatively, the consumption of one liter of orange juice containing 71 mg of CyG in fasting human volunteers resulted in around 490 nM of PCA in serum after 2 h, accounting for over 70% of ingested CyG (1). Another research has reconfirmed that PCA is the major phenolic compound in blood after the consumption of cranberry juice (26). These indicate that PCA has a high bioavailability, highlighting its nutritional value and therefore the significance of investigating the bioactivity of PCA. In the present study, incubation with 100 nM PCA could reverse endothelial dysfunction in aortas from *db/db* diabetic mice, suggesting the concentration of PCA needed to exert protection against endothelial dysfunction in diabetic aortas is physiologically achievable by consuming a regular diet.

To the best of our knowledge, we are the first research group to prove that direct incubation with physiological concentration of PCA exerts significant protective effects on endothelial function against diabetic damage. A previous study has proposed that oral PCA treatments could restore the blood pressure, serum nitrate/nitrite ratio, as well as plasma anti-oxidative enzyme levels in streptozotocin-induced diabetic rats (4). Another research group had added a set of functional data to indicate that oral PCA administration could improve insulin-induced vasorelaxation, with enhanced serum anti-oxidative enzyme activities and serum nitrate/nitrite concentration in spontaneously hypertensive rats (5). On top of these systemic effects, the present results have clearly demonstrated direct effects of PCA on the protection of endothelial function against diabetes.

Aortas are one of the arteries most susceptible to endothelial dysfunction in diabetes (27, 28). Whether PCA improves endothelial functions was thus first evaluated in the *db/db* diabetic mouse aortas using wire myograph. In our results, improvement in endothelium-dependent relaxation (EDR) was observed in both male and female *db/db* mouse aortas with PCA treatment. Intriguingly, PCA was able to rescue the EDR in the aortas from the female *db/db* diabetic mice to a level comparable to their *db/m^+^* counterparts, whereas the EDR for the male, despite being significantly reversed, was only up to a half of the extent of the impairment. This may be because the gap between the EDR in *db/db* and *db/m^+^* was smaller in female than in male, which may in turn be due to a reduced vessel wall thickness and collagen content in male *db/db* mouse aortas (29). However, the mean difference between *db/db* with and without PCA treatments was similar in male and female. Our data suggest that PCA can protect the endothelial function in *db/db* mouse aortas in both genders, yet whether there is any additional sexual difference in the response to PCA may require further confirmation.

The present study unveiled that IL-1β-induced EDR impairment and ROS overproduction in aortas from control mice could be reversed by PCA. As the key regulator of vascular homeostasis, endothelial cells are highly sensitive to but also easily damaged by changes in vascular risk factors, such as ROS, LPS and pro-inflammatory cytokines, in blood (30). In addition to the blood glucose level, the serum pro-inflammatory cytokine levels are also heightened in diabetes (31, 32). Research has found that EDR impairment, ROS and pro-inflammatory overproduction in endothelium cannot be induced by hyperglycemia alone, but only in a pre-existing pro-inflammatory environment (18). The pro-inflammatory state built by the elevated circulating levels of pro-inflammatory cytokines, including IL-1β, IL-6 and TNF-α, in diabetes exactly provides the pre-requisite for endothelial dysfunction, accounting for CVDs being the most common cause of morbidity and mortality in diabetic patients (33). In particular, the classic pro-inflammatory cytokine IL-1β was found to be a critical contributor in diabetic endothelial dysfunction (34). Upregulated level of IL-1β has been reported to be induced by hyperglycemia in human aortic endothelial cells (35), myocardium, macrophages and hearts from diabetic rats. Indeed, antagonizing the receptor for IL-1β, interleukin-1 receptor (IL-1R), was suggested to be promising in modulating the chronic pro-inflammatory status in diabetic patients (36). Apart from the previously reported anti-oxidative effects, our present study implemented the IL-1β-induced pro-inflammatory model to clearly demonstrate the anti-inflammatory effect of PCA that IL-1β-induced EDR impairment and ROS overproduction in aortas from control mice could both be reversed by the incubation with 100 nM PCA.

Based on the functional data, we hypothesized that PCA may protect endothelial function against inflammation through enhancing eNOS activation. Since deficient eNOS phosphorylation in brain microvascular endothelial cells (BMECs) is associated to increased stroke size and eNOS is a therapeutic target for cerebrovascular diseases (37, 38), whether PCA could improve endothelial function in mBMECs was evaluated. In agreement with the previous data on aortic rings, the ROS level exaggerated by IL-1β in endothelial cells was alleviated by PCA treatment. However, since DHE staining was the only method implemented to measure the ROS level, the identities and sources of the oxidative species could not be clarified and would need further assessment. Furthermore, PCA suppressed the IL-1β-induced gene expression of pro-inflammatory cytokines and oxidative inducible nitric oxide synthase (iNOS), while promoting that of endothelial nitric oxide synthase (eNOS) and the novel vasoprotective gene uncoupling protein 1 (UCP1) (39, 40). As UCP1 has recently been found to play important roles in ROS production in diabetes (41), we hypothesized that PCA treatment may inhibit ROS production through upregulating UCP1, which would need further validation in future studies. The regulating effects on inflammation by PCA were reinforced by western blotting, which showed a dose-dependent downregulation of monocyte chemoattractant protein-1 (MCP1) with enhanced eNOS and Akt phosphorylation. Nevertheless, future investigations using arteries from other positions, such as skeletal muscle feed arteries and mesenteric resistance arteries, as well as endothelial cells from the other arteries would be of interest to explore and reinforce the effects of PCA on endothelial function.

The present data showed that PCA dose-dependently augmented Akt phosphorylation suppressed by IL-1β. Akt is a crucial regulator of endothelial cell functions (42). Along with regulating endothelial cell migration, survival and tube formation, Akt also maintains nitric oxide (NO) production and thus the homeostasis in endothelial cell through direct phosphorylation of eNOS (43). In type 2 diabetes, however, Akt phosphorylation, as well as the efficiency for activated Akt to phosphorylate eNOS are reduced (44, 45). Impairment of this Akt/eNOS pathway contributes to diabetic endothelial dysfunction (46, 47). On the other hand, treatments enhancing Akt activation have been suggested to be beneficial to vascular health by promoting eNOS activity (48, 49). In the present study, in the presence of Akt inhibitor IV, phosphorylated Akt was low in all three groups, indicating that the phosphorylation of Akt was inhibited. Although phosphorylation of eNOS was hindered by the inhibition of Akt phosphorylation, it was further suppressed by IL-1β and failed to be reversed by PCA. Moreover, MCP1, VCAM1, ICAM1 and iNOS exaggerated by IL-1β remained high with PCA treatment when Akt phosphorylation was inhibited. These suggest that effect of PCA on endothelial cells was mediated through Akt signaling.

Last but not least, more questions are awaited to be answered: whether PCA also acts through other signaling pathways, such as AMPK, ERK, PKA, which also affect eNOS activity; whether PCA would also affect the dimerization and phosphorylation sites of eNOS other than the Ser^1177^ examined in the current study; as well as whether PCA also have other effects on smooth muscle cells. Solving these questions would be needed to complete the picture of the effects of PCA on vascular functions.

## 5. Conclusion

In summary, we have demonstrated that PCA possesses a strong anti-inflammatory activity on endothelial cells and can ameliorate the endothelial dysfunction through enhancing Akt/eNOS pathway. Although how exactly PCA is engaged in the signaling pathway and whether gender differences exist in its effects require further investigations, the current findings suggest that acquisition of PCA from daily diets may be beneficial to the vascular health in diabetic patients.

## Data availability statement

The original contributions presented in this study are included in the article/Supplementary material, further inquiries can be directed to the corresponding authors.

## Ethics statement

The animal study was reviewed and approved by Animal Experimentation Ethics Committee (AEEC), CUHK.

## Author contributions

CC conducted the experiments, collected and analyzed data, and prepared the draft manuscript. YC provided the critical comments on experimental design and data interpretation. KM and FL offered the technical support. HZ and QN developed the ideas and provided the critical comments on data interpretation. WW supervised the research and provided critical review on the manuscript. Z-YC developed the concept and supervised the research. All authors read and approved the final manuscript.
