# Supplementary figures and images for: Physiological concentration of protocatechuic acid directly protects vascular endothelial function against inflammation in diabetes through Akt/eNOS pathway
Source: Front Nutr. 2023 Mar 21;10:1060226. doi: 10.3389/fnut.2023.1060226 (PMC10070727; doi:10.3389/fnut.2023.1060226)

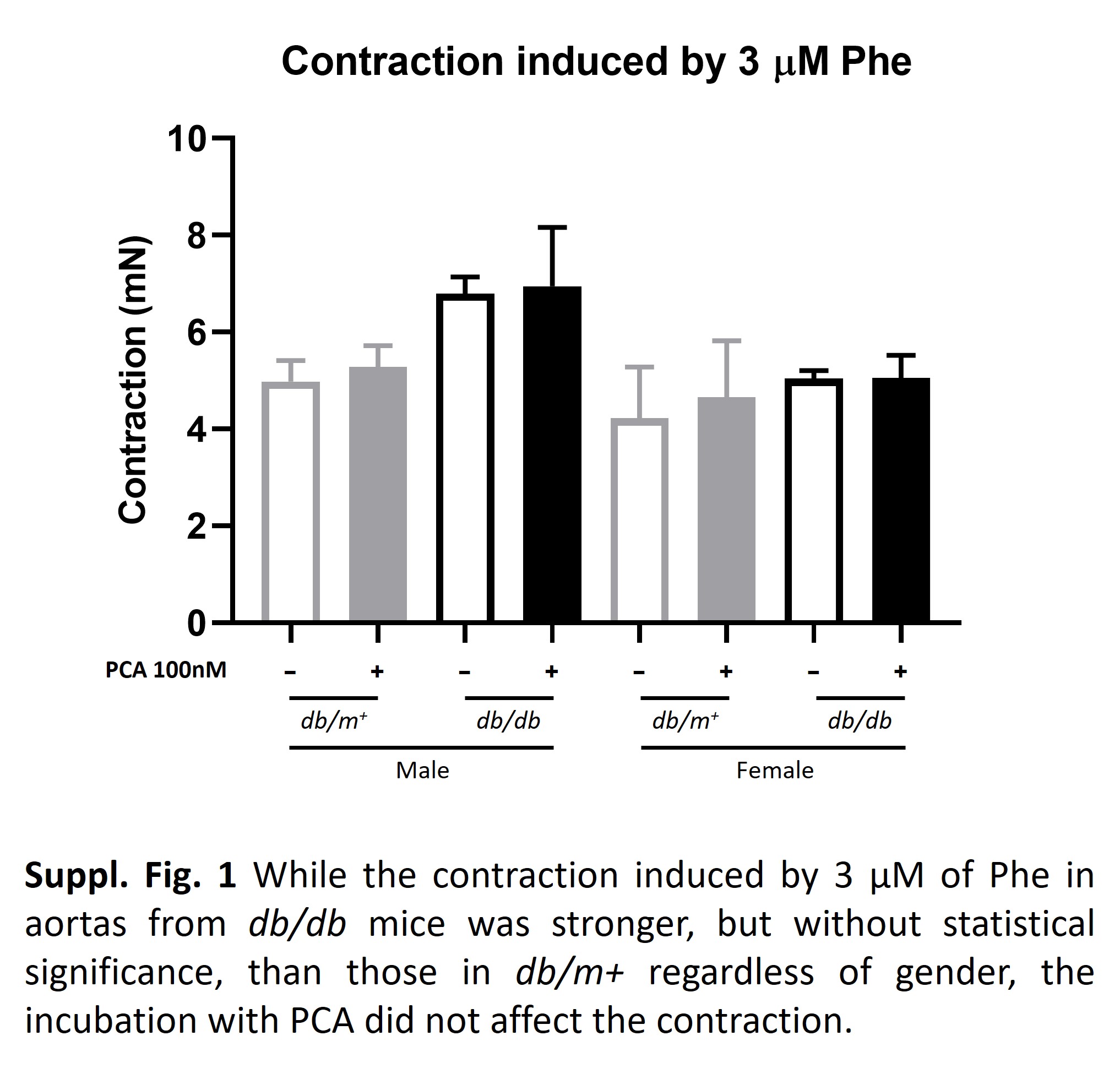

Supplement: Supplementary file 1 [file Image_1.JPEG]

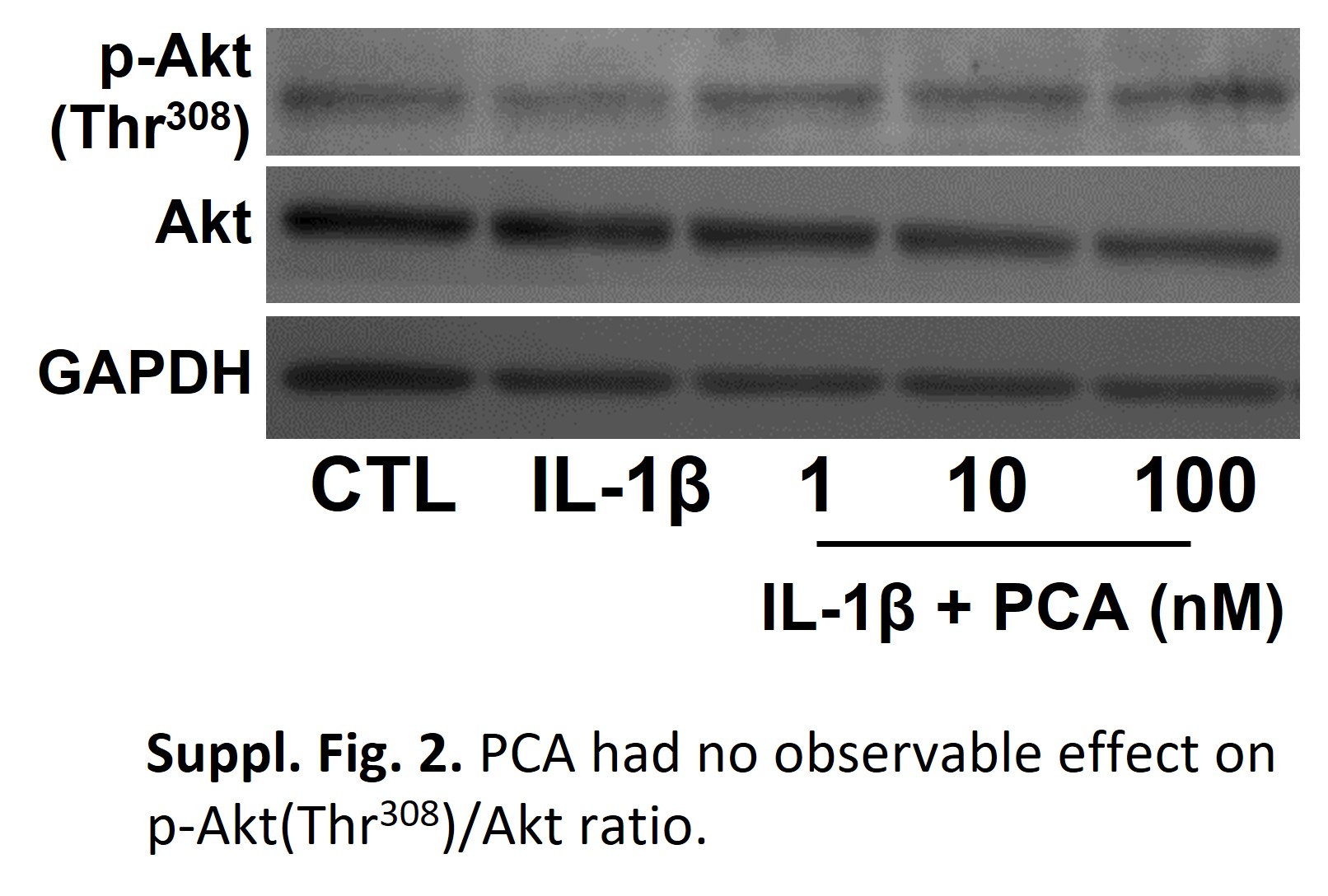

Supplement: Supplementary file 2 [file Image_2.jpg]
